# Evolution of the Ainu Language in Space and Time

**DOI:** 10.1371/journal.pone.0062243

**Published:** 2013-04-26

**Authors:** Sean Lee, Toshikazu Hasegawa

**Affiliations:** Department of Cognitive and Behavioral Science, Graduate School of Arts and Sciences, University of Tokyo, Tokyo, Japan; IPATIMUP (Institute of Molecular Pathology and Immunology of the University of Porto), Portugal

## Abstract

Languages evolve over space and time. Illuminating the evolutionary history of language is important because it provides a unique opportunity to shed light on the population history of the speakers. Spatial and temporal aspects of language evolution are particularly crucial for understanding demographic history, as they allow us to identify when and where the languages originated, as well as how they spread across the globe. Here we apply Bayesian phylogeographic methods to reconstruct spatiotemporal evolution of the Ainu language: an endangered language spoken by an indigenous group that once thrived in northern Japan. The conventional dual-structure model has long argued that modern Ainu are direct descendants of a single, Pleistocene human lineage from Southeast Asia, namely the Jomon people. In contrast, recent evidence from archaeological, anthropological and genetic evidence suggest that the Ainu are an outcome of significant genetic and cultural contributions from Siberian hunter-gatherers, the Okhotsk, who migrated into northern Hokkaido around 900–1600 years ago. Estimating from 19 Ainu language varieties preserved five decades ago, our analysis shows that they are descendants of a common ancestor who spread from northern Hokkaido around 1300 years ago. In addition to several lines of emerging evidence, our phylogeographic analysis strongly supports the hypothesis that recent expansion of the Okhotsk to northern Hokkaido had a profound impact on the origins of the Ainu people and their culture, and hence calls for a refinement to the dual-structure model.

## Introduction

Patterns of linguistic variation among individuals often carry the signature of a speech community's demographic past. Accumulating evidence indicates that languages evolve by a process of descent with modification and they form into distinct families in a manner similar to their speakers forming into different ethnic groups through evolutionary history [1]–[3]. The intertwined history between languages and their speakers appears most vividly in the areas that experienced large-scale population expansions, often driven by agricultural intensification and cultural innovation since the end of the last Ice Age [4]. Recent empirical evidence supporting this phenomenon includes a range of language phylogenies reconstructed with computational methods [5]–[8].

While the computational phylogenetic methods have been fruitful in shedding new light on language evolution and the speakers’ prehistory, their application has been focused mainly on inferring temporal and sequential aspects. As a result, inferences about the homeland or geographic diffusion pattern often relied on heuristic approaches such as locating a monophyletic outgroup and formulating post-hoc diffusion scenarios from the branching order. Recent progress in phylogenetic methods is, however, producing innovative ways to embed phylogenetic inference in a geographical context, and allow us to explicitly estimate both temporal and spatial aspects of evolution while accounting for phylogenetic uncertainty [9]–[11]. In this paper, we adopt these methodological innovations and directly reconstruct spatiotemporal evolution of the Ainu language: a nearly extinct language spoken by indigenous people of Japan whose origins remain obscure.

Considerable debate surrounds the apparent incompatibility between the conventional model of human prehistory for the Japanese islands and the emerging evidence from modern archaeology, anthropology and genetics. For several decades, the dual-structure model [12] has posited that similarities in dental [13] and cranial features [14] between the Ainu people and Southeast Asians meant that the Ainu ancestry originated in Southeast Asia around 10700 years before present (BP) [15]. Similarly, reconstructed proto-Ainu lexicons have also been suggested to share some similarities with proto-Austroasiatic lexicons [16]. Therefore, the Ainu have long been thought to be direct descendants of a single ancient Southeast Asian lineage, the Jomon, and have remained isolated from neighboring populations throughout the Holocene. However, recent evidence from genetic [17], [18], morphological [19], [20], and cultural studies [21] are beginning to suggest that the Okhotsk people, a hunter-gatherer group from the Amur river basin, migrated into northern Hokkaido around 900–1600 BP bringing significant genetic and cultural contributions to the preexisting Jomon, and subsequently gave rise to modern Ainu people as well as their culture. In essence, this ‘Okhotsk expansion scenario’ suggests that, far from being direct descendants of a single ancient human lineage that had no contact with the rest of the world, the Ainu and their culture are the outcome of a recent population expansion into northern Hokkaido.

**Figure 1:**
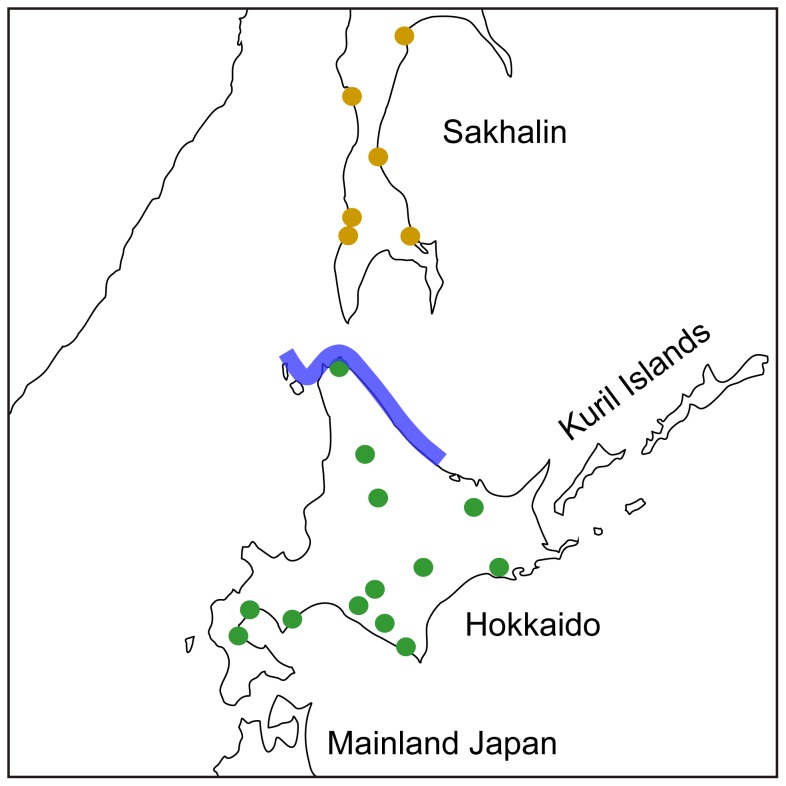
Map of the Ainu language varieties. Colored circles represent two major subgroups (Green-Hokkaido; Yellow-Sakhalin). Blue bar in the center indicates the main area of the Okhotsk settlement.

If we accept premises (i) population expansions often leave its signature in the patterns of linguistic variation and (ii) the cultural flow from the incoming Okhotsk people had a profound impact on the language, then we can reason that spatiotemporal reconstruction of the Ainu language evolution might allow us to test the plausibility of the Okhotsk expansion scenario for the Ainu origin, and examine whether or not the dual-structure model should be modified to accommodate the Okhotsk expansion scenario. Accordingly, we predicted that if the scenario were correct, then the estimated root age of the Ainu varieties should coincide with 900–1600 BP, and their geographic distribution should be the end result of expansion from northern Hokkaido, where the gene and cultural flows from the Okhotsk to Jomon is likely to have taken place (blue bar in Figure 1). It should be noted that even if the patterns of Okhotsk expansion were correct, the specific processes of language change could be interpreted in two ways (see Discussion for more details). Following the line of reasoning above, we also predicted that if the scenario were incorrect, then the Ainu language diffusion should conform to the conventional scenario and spread northward from southern Hokkaido with the root age being at least several thousand years older than 1600 BP but not beyond 10000 BP, which is the current methodological limit for tracing language ancestry.

## Materials and Methods

The data consist of 19 geocoded lists of 200 basic vocabularies (Figure 1; for a full list of sites, see Figure S1) compiled by Hattori and Chiri during 1950s [22], when there was still a rich linguistic diversity among the Ainu people. The basic vocabularies are a set of words transmitted vertically from one generation to the next [23], thereby preserving evolutionary signal required for reconstructing phylogenetic history [3], [24]. Nevertheless, one could argue that the 19 varieties that we analyze here are dialects of the Ainu language and if one supposes that only languages, not dialects, constitute representative units of analysis, then using these varieties implies that the resulting tree may potentially depict a confusing branching pattern with excessive detail, or even fail to recover the actual subdivisions of the speech community [25].

We do not, however, consider this to be a major obstacle for reconstructing Ainu language evolution for three reasons: (i) a natural model of language evolution that we use here is known to be robust against reasonable levels of noise (i.e., up to 20% of horizontal transfer per 1000 years) [26], (ii) if we define languages as groups of tongues that are mutually unintelligible in a manner similar to biologists defining species as groups of animals that cannot interbreed [27], then Swadesh’s criterion of mutual intelligibility (i.e., any two languages being mutually unintelligible if they share less than 90% of their basic vocabularies with each other [28]) and a matrix of pair-wise cognate similarities of the Ainu varieties [22] allow us to estimate that any one of the varieties would be able to communicate with the rest only about 18% at a time, meaning that the majority of the 19 varieties can actually be considered languages in their own right and (iii) we used SPLITSTREE4 [29] to estimate tree-likeness of the Ainu phylogeny [30], [31] and obtained the average delta score = 0.25 and *Q*-residual score = 0.01, both indicating that the evolution of Ainu lexicons was reasonably tree-like, and hence suitable for phylogenetic analysis (to put this in perspective, the tree-likeness scores calculated from a subset of 12 Indo-European languages have similar scores as our 19 Ainu varieties with the average delta score = 0.23 and *Q*-residual score = 0.03 [31]). These observations provide us confidence that the data should carry robust evolutionary signal and the 19 Ainu varieties are appropriate units of analysis for the current purpose.

Cognate judgments, a process of revealing shared ancestry among lexicons, are carried out by identifying systematic correspondences in phonetic structure and meaning [25]. For our analyses, we adopted the cognate judgments made by the two linguists who compiled the data [22]. The cognate sets were encoded into binary states indicating presence ('1') or absence ('0') of a cognate, which resulted in 19×350 matrix.

We used BEAST [32] for all analyses because it allows us to reconstruct phylogenies without specifying an *a priori* outgroup. Continuous random walk model we use in this paper [9], [33] is a Bayesian expansion of Brownian diffusion model developed in a maximum-likelihood framework [34]. In general, a Brownian diffusion model aims to estimate the vectors of latitudes and longitudes of internal nodes (i.e., common ancestors of extant languages) on a continuous surface, in which increments are independent and normally distributed with a mean centered on zero with variance that scales linearly in time, meaning that diffusion processes are assumed to be homogeneous over time and space. This can be unrealistic as many geographic features (e.g., mountains and rivers) can influence the rate of spread for each branch. Bayesian continuous diffusion model we adopt here effectively overcomes this limitation by relaxing the Brownian process: borrowing ideas from uncorrelated relaxed clock models [35], the method models branch-specific dispersal processes with the diffusion rate scalar in each branch being drawn independently and identically from a range of parametric distributions. Distributions used in our analyses are (i) Cauchy distribution that has fat tails accommodating long distance dispersals [36], (ii) gamma distribution that accommodates infinite variance in a manner similar to Lévy flight models [37] but without enforcing power-law tail behavior, and (iii) lognormal distribution that allows even greater degree of rate variability [35]. In order to make our geographic inference more realistic, we sampled the root and node locations only from the land by assigning a prior probability of zero to the water [11].

In addition, we compared the degree of model-fit between relaxed and strict clocks [35]. Temporal scale of phylogenies was calibrated using a probabilistic prior taken from well-attested evidence that modern Ainu expanded into Sakhalin around 15th century [38], [39]: a normally-distributed prior with a mean of 500 BP with its 95% of the distribution incorporating 200 years of uncertainty. For all analyses, we applied a stochastic Dollo model with a correction for ascertainment bias [40] and a Bayesian skyline tree prior [41]. We chose the best model by comparing Bayes Factors (BF) [42].

## Results

**Figure 2:**
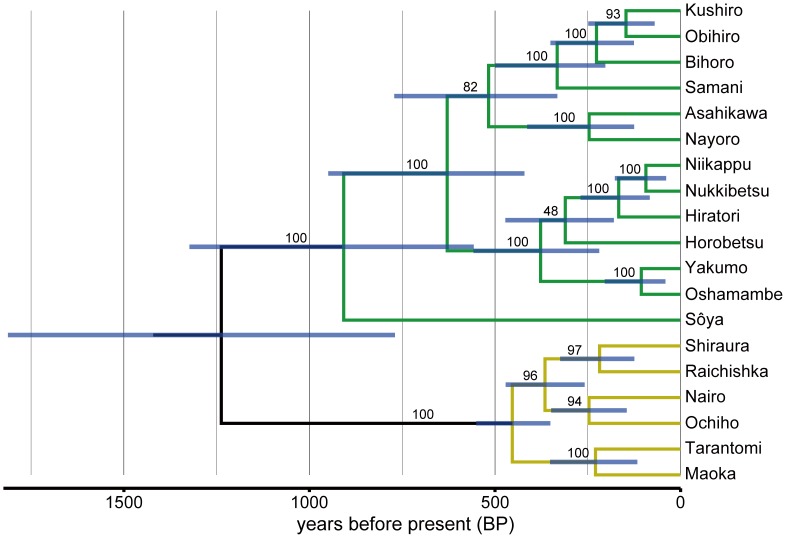
Maximum clade credibility tree of 19 Ainu language varieties. Colored branches represent two major subgroups (Green-Hokkaido; Yellow-Sakhalin). All node heights are scaled to match the posterior median node heights with bars indicating 95% HPD intervals of the estimated ages. The value on each branch is the posterior probability, showing the percentage support for the following node.

**Figure 3:**
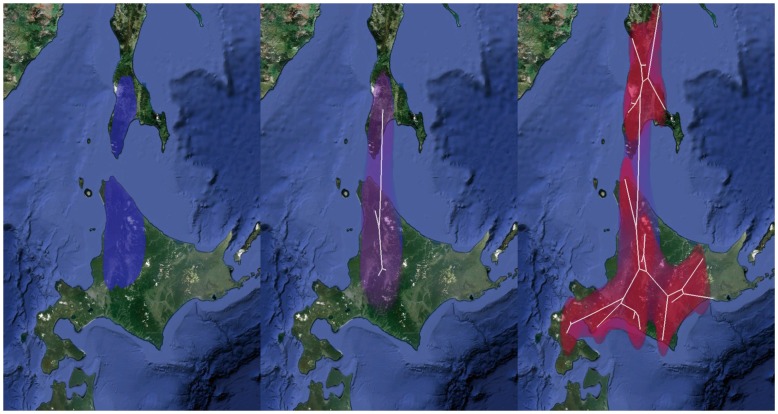
Inferred origin and diffusion of the Ainu language varieties in natural time scale. Color gradient of the polygons (80% HPD) indicates relevant age of the diffusion [Blue-older (1288 BP); Red-more recent (50 BP)]. White lines represent the phylogeny projected onto the surface. Image sources: © 2012 Google Earth; © 2012 Cnes/Spot Image; © 2012 TerraMetrics.

Based on BF tests among diffusion models and evolutionary clock models, we chose the relaxed clock with gamma-distributed diffusion as the best model (Table S1). Figure 2 shows the summary of time-dated maximum clade credibility trees for 19 Ainu language varieties. Assuming that the patterns of linguistic diversity is shaped by the demographic dynamics of speakers, we predicted that if the recent evidence supporting the Okhotsk expansion scenario were correct, then the estimated root age should overlap with 900–1600 BP. The estimated root age of the Ainu language across post-burn-in trees has a median of 1288 BP [mean: 1323 BP; 95% Highest Posterior Density (HPD): 820–1862 BP], in strong agreement with the prediction. We also predicted that if the hypothesized scenario were correct, then the current distribution of 19 Ainu language varieties should be the end result of diffusion from northern Hokkaido; otherwise, we would observe northward expansion from southern Hokkaido, conforming to the conventional dual-structure model. Figure 3 (also in Animation S1) shows that the estimated diffusion pattern in natural time scale [43] is in clear agreement with the prediction, with the estimated homeland being in northern Hokkaido. Both the diffusion pattern and root time were consistent across all models we excluded based on BF tests.

In order to examine the robustness of our phylogeographic inferences, we carried out two additional tests. Firstly, we tested the strength of support for northern Hokkaido origin (i.e., the Okhotsk expansion scenario) over southern Hokkaido origin (i.e., the dual-structure model) by directly calculating BF: we divided Hokkaido into two broad regions of north and south at the centroid of Hokkaido, and estimated BF by comparing the posterior to prior odds ratio of observing potential homeland in either one of the two regions. In agreement with our results, we obtained substantial support (BF = 7.5) for northern Hokkaido being the homeland of the Ainu. Secondly, we investigated whether or not our results are statistical artifacts of the diffusion model falling into the center of language mass regardless of the data: we randomly reassigned the locations of 19 Ainu varieties to the data for fifty times, and then obtained 90% HPDs for all possible root locations (Figure S2). From this exercise, we observed that the absence of true signal could cause the estimated homeland to be as south as mainland Japan or as north as Sakhalin. This observation clearly demonstrates that our results are valid estimations based on true phylogeographical signal. Conversely, this also suggests that if the data contained signal indicating northward diffusion, or any other direction, our methods would have reconstructed it accordingly.

We acknowledge, however, that a well-established subgroup of the Ainu language, namely the Kuril, is absent from our data. This is because the Kuril had become extinct by the time the data were collected, and the Kuril lexicons seem to be available only through sketchy records scattered around the literature. For this reason, we currently have little information about the Kuril. If the point in time that the Kuril diverged from other varieties turns out to be much deeper, then the resulting divergence time and diffusion pattern may differ significantly from the current results. The search for a more complete set of data is, therefore, a direction that should be prioritized for further evaluation of our conclusion.

## Discussion

In this paper, we reconstructed spatiotemporal evolution of 19 Ainu language varieties, and the results are in strong agreement with the hypothesis that a recent population expansion of the Okhotsk people played a critical role in shaping the Ainu people and their culture. Together with the recent archaeological, biological and cultural evidence, our phylogeographic reconstruction of the Ainu language strongly suggests that the conventional dual-structure model must be refined to explain these new bodies of evidence. The case of the Ainu language origin we report here also contributes additional detail to the global pattern of language evolution, and our language phylogeny might also provide a basis for making further inferences about the cultural dynamics of the Ainu speakers [44], [45].

We recognize that there are also some evidence that the Jomon people, one of the two ancestral populations of the Ainu, may have descended from Northeast Asia rather than Southeast [46], [47], thereby questioning the validity of dual-structure model on a greater time scale. Unfortunately, the scope of our results presented here have little bearing on the larger question of the Jomon prehistory because the linguistic traces of this process may have been wiped out by the recent rise of the Ainu as our results indicate. Regardless of what further research reveals about the Jomon ancestry, however, we argue that the evidence for the Okhotsk expansion scenario should remain valid, and therefore any future models of deeper historical process for the Japanese islands must properly account for the recent northern Hokkaido origin of the Ainu. With this respect, we suggest that the most effective way of shedding light on the deeper history of the Jomon, or historical processes of any other regions, is to synthesize different lines of evidence from archaeology, biology and culture, and triangulate them to obtain a rigorous analytic framework [48] rather than relying on a single line of evidence.

If our inferences are correct, then the recent Okhotsk expansion scenario for the Ainu origin leads us to a new question: what historical factors drove the Okhotsk people to migrate from the Amur river basin to Hokkaido and give rise to the Ainu? It is now clear that early farming populations went through similar processes due to agricultural intensification and cultural innovation [4] but the Okhotsk people were hunter-gatherers, not farmers. While not resolving this question directly, Hudson [49] provides a comprehensive model of the Okhotsk socio-environmental conditions that allows us to sketch out a possible scenario: (i) the diet of the Okhotsk people relied heavily on marine mammal products and (ii) the time in which the Okhotsk expansion occurred seems to be characterized by dramatic climate changes, beginning with a cold sea-ice stage between 1300–1800 BP followed by a warmer open-ocean stage. Based on these observations, we speculate that the Okhotsk expansion may have been opportunistic in nature: the sea-ice condition in the early stage probably resulted in increased area for exploiting marine mammals as well as convenient routes for exploring new territory, thereby leading to the migration into Hokkaido. The drastic climate change in the later stage, however, may have deteriorated the hunting conditions for the Okhotsk with rapid break up of sea-ice, which may also have necessitated increased reliance on other types of food source, and hence causing a greater degree of niche overlap with the preexisting Jomon population. The end result was probably the admixture of the two populations, followed by the rise of a new ethnolinguistic group, namely the Ainu.

If we accept a view that transmission of language may be gender-specific [50]–[52], then we are able to formulate at least two hypotheses for the specific processes of the Ainu language origin. Because Y-chromosome haplogroup D is thought to represent Jomon male ancestry, the predominance of that particular haplogroup in the Ainu (75–87.5%) implies that the majority of Ainu male ancestry is from the Jomon [53], [54], whereas a heavy mixture of mtDNA haplogroups indicates that a significant proportion of the Ainu female ancestry is from the Okhotsk (excluding 35.3% of mtDNA haplogroups that the Ainu share with other neighboring populations, 39.4% of the remaining female heritage is shared exclusively with the Okhotsk and the rest is a mixture of both Jomon and Okhotsk [18], [47], [54]). If we thus assume male-specific language transmission for the Ainu, the first hypothesis for the processes behind the Ainu language origin could be that proto-Ainu arose from a large number of Jomon males who intermarried with Okhotsk females in northern Hokkaido, and subsequently spread to the rest of region. Similarly, if we assume that the transmission of Ainu language corresponds with female ancestry, the second hypothesis could be that proto-Ainu was spoken by the incoming Okhotsk females who merged with the preexisting Jomon males. Based on these observations, we propose that one potential way of understanding how language change occurred for the Ainu is to estimate which gender was more influential when early Ainu people established family membership. This may be carried out indirectly by revealing the signature of historical post-marital residence pattern via estimating the degrees of genetic variation in their Y-chromosome and mtDNA [55] as well as reconstructing ancestral post-marital residence rules from regional cultural variation [56]. Investigating which model of language change [57] is relevant to the Ainu is a direction that deserves more attention, and acquiring an accurate description of how language change occurred for the Ainu would allow us to make further inferences about the deeper history of the human lineage that once thrived in northern Japan.

Languages rise and fall, and so do the communities who speak them. Although significant progress has been made in recent years, we are still far from thoroughly understanding why languages are so deeply related to the fates of their speakers or how the process unfolds through evolutionary history. These are perhaps some of the most challenging questions in human sciences, and a complete understanding of this complex phenomenon might thus be reached only with further methodological innovations as well as more language data from around the world. But as we demonstrate in this paper, a combination of spatiotemporal reconstruction of language evolution and synthesis of several different historical evidences is probably one of the most promising methodologies that can further illuminate the process and consequence of this fascinating phenomenon.

## Supporting Information

Figure S1 **Full list of the Ainu language varieties.** Colored circles represent subgrouping (Green-Hokkaido; Yellow-Sakhalin).
(TIF)

Figure S2 **Ninety percent highest probability density obtained from fifty random reassignments of location coordinates to the tips of phylogeny.** This demonstrates that our results are not statistical artifacts of the diffusion model returning to the center of language mass. For all analyses, we applied an arbitrary root calibration consisting of a normal distribution with the mean of 1500 BP and the standard deviation of 400 years.
(TIFF)

Table S1 **Log-marginal likelihoods estimated from all models fitted to data.** The model with a relaxed clock and gamma-distributed random walk model shows the best fit with the highest log-marginal likelihood.
(DOCX)

Animation S1 **Animated origin and diffusion of the Ainu language varieties in natural time scale.** Color gradient of the polygons (80% HPD) indicates relevant age of the diffusion [Blue-older (1288 BP); Red-more recent (50 BP)]. White lines represent the phylogeny projected onto the surface. Image sources: © 2012 Google Earth; © 2012 Cnes/Spot Image; © 2012 TerraMetrics.
(MOV)

Analysis S1 **NEXUS and BEAST input files for full details of the analysis.**
(ZIP)
