# Supplementary figures and images for: Evolution of the Ainu Language in Space and Time
Source: PLoS One. 2013 Apr 26;8(4):e62243. doi: 10.1371/journal.pone.0062243 (PMC3637396; doi:10.1371/journal.pone.0062243)

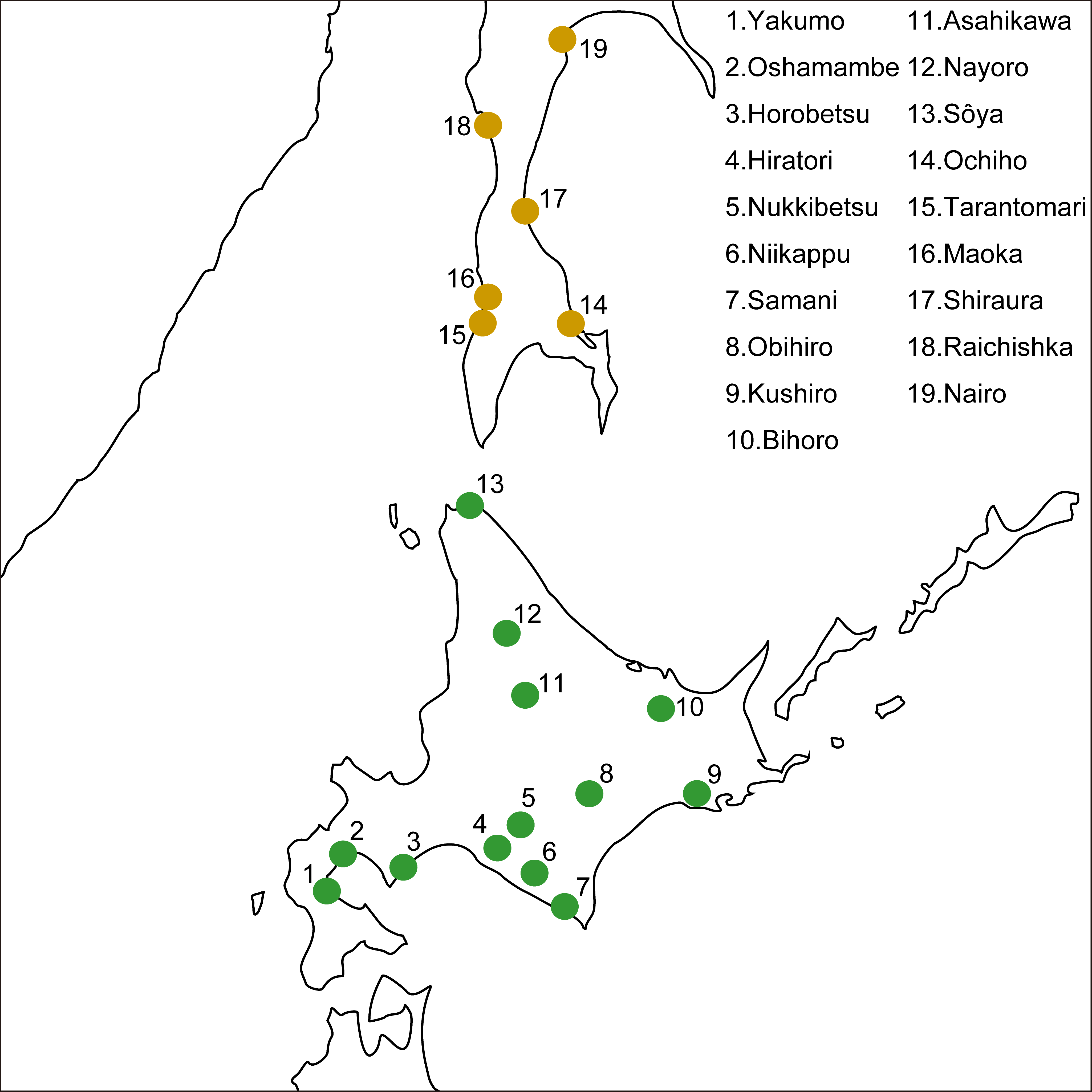

Supplement: Figure S1 — Full list of the Ainu language varieties. Colored circles represent subgrouping (Green-Hokkaido; Yellow-Sakhalin). (TIF) [file pone.0062243.s001.tif]

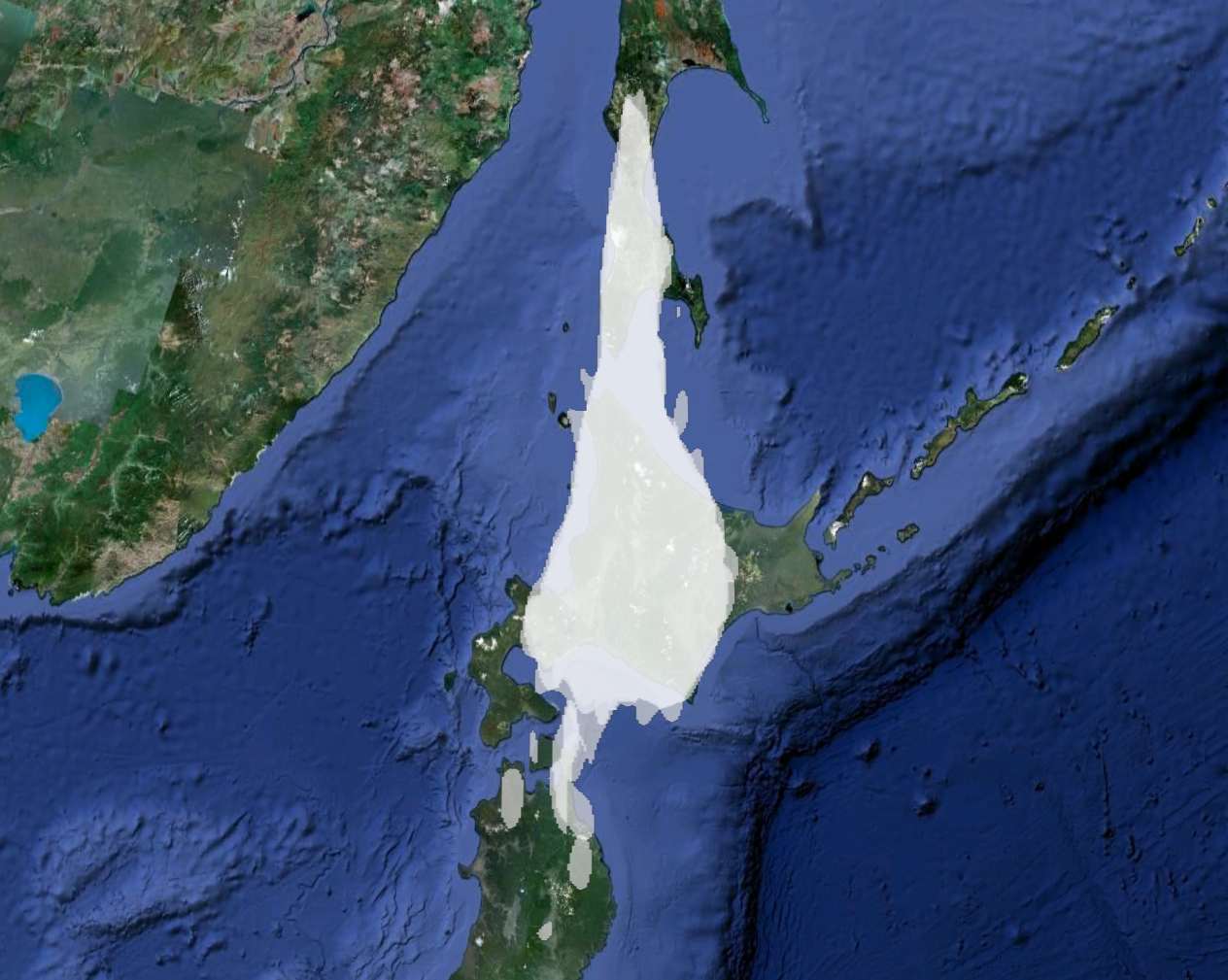

Supplement: Figure S2 — Ninety percent highest probability density obtained from fifty random reassignments of location coordinates to the tips of phylogeny. This demonstrates that our results are not statistical artifacts of the diffusion model returning to the center of language mass. For all analyses, we applied an arbitrary root calibration consisting of a normal distribution with the mean of 1500 BP and the standard deviation of 400 years. (TIFF) [file pone.0062243.s002.tiff]
