# Supplementary material for: Evolution of the Ainu Language in Space and Time
Source: PLoS One. 2013 Apr 26;8(4):e62243. doi: 10.1371/journal.pone.0062243 (PMC3637396; doi:10.1371/journal.pone.0062243)
Supplement: Table S1 — Log-marginal likelihoods estimated from all models fitted to data. The model with a relaxed clock and gamma-distributed random walk model shows the best fit with the highest log-marginal likelihood. (DOCX) [file pone.0062243.s003.docx]

Table S1. Log-marginal likelihoods estimated from all models fitted to data.

| **Diffusion models** | **Relaxed clock** | **Strict clock** |
| --- | --- | --- |
| Homogeneous Brownian | -2364.20 | -2368.69 |
| Cauchy | -2367.58 | -2375.33 |
| Gamma | -2362.93 | -2369.06 |
| Lognormal | -2365.46 | -2374.05 |

The model with a relaxed clock and gamma-distributed random walk model shows the best fit with the highest log-marginal likelihood.
